# Organotin compounds in surface sediments of the Southern Baltic coastal zone: a study on the main factors for their accumulation and degradation

**DOI:** 10.1007/s11356-013-2115-x

**Published:** 2013-09-12

**Authors:** Anna Filipkowska, Grażyna Kowalewska, Bruno Pavoni

**Affiliations:** 1 Marine Pollution Laboratory, Institute of Oceanology, Polish Academy of Sciences, ul. Powstańców Warszawy 55, 81-712 Sopot, Poland; 2 Department of Environmental Sciences, Informatics and Statistics, University of Venice, Calle Larga S. Marta 2137, 30123 Venice, Italy

**Keywords:** Tributyltin, Organotins, Sediment, Baltic Sea, Vistula Lagoon, Szczecin Lagoon

## Abstract

Sediment samples were collected in the Gulf of Gdańsk, and the Vistula and Szczecin Lagoons—all located in the coastal zone of the Southern Baltic Sea—just after the total ban on using harmful organotins in antifouling paints on ships came into force, to assess their butyltin and phenyltin contamination extent. Altogether, 26 sampling stations were chosen to account for different potential exposure to organotin pollution and environmental conditions: from shallow and well-oxygenated waters, shipping routes and river mouths, to deep and anoxic sites. Additionally, the organic carbon content, pigment content, and grain size of all the sediment samples were determined, and some parameters of the near-bottom water (oxygen content, salinity, temperature) were measured as well. Total concentrations of butyltin compounds ranged between 2 and 182 ng Sn g^−1^ d.w., whereas phenyltins were below the detection limit. Sediments from the Gulf of Gdańsk and Vistula Lagoon were found moderately contaminated with tributyltin, whereas those from the Szczecin Lagoon were ranked as highly contaminated. Butyltin degradation indices prove a recent tributyltin input into the sediments adjacent to sites used for dumping for dredged harbor materials and for anchorage in the Gulf of Gdańsk (where two big international ports are located), and into those collected in the Szczecin Lagoon. Essential factors affecting the degradation and distribution of organotins, based on significant correlations between butyltins and environmental variables, were found in the study area.

**Electronic supplementary material:**

The online version of this article (doi:10.1007/s11356-013-2115-x) contains supplementary material, which is available to authorized users.

## Introduction

Organotin compounds (OTs), due to their toxicity, persistence, and wide utilization during the last 60 years, are chemicals of great environmental concern. They have been used in the plastic industry, in agriculture, and also as antifouling ingredients in paints. The last application is the most important for the marine environment, because some organotins (most of all tributyltin (TBT) and triphenyltin (TPhT)) have been used for many years to prevent the settlement and growth of aquatic organisms on ship hulls, fishing nets or cages, oil rig supports, and different tools used in seawater. These kinds of paints have shown a special effectiveness, but proved to be toxic for aquatic life (Alzieu 1998; Champ 2000; Hoch 2001).

TBT and TPhT can disrupt the endocrine system of aquatic organisms at very low concentrations. TBT is extremely toxic to algae, zooplankton, and the larval stages of some fish species, but in particular to mollusks. This compound causes the appearance of male sexual characters in the females (known as imposex), disorders in the reproduction and shell calcification anomalies (Alzieu 2000; Antizar-Ladislao 2008; Rüdel 2003). TPhT, although apparently less toxic, also poses a hazard to aquatic life (Hoch 2001). Both TBT and TPhT have shown high environmental persistence and the ability to transfer along the trophic chains (Strand and Jacobsen 2005; Veltman et al. 2006). They are recognized as one of the most hazardous substances that have been released into the marine environment deliberately (HELCOM 2010).

Degradation of OTs, which consists in a progressive loss of organic groups from the Sn cation, can be caused by various processes including photolysis and chemical and biological cleavage. In the sea, TBT and TPhT, due to their hydrophobic properties, tend to adsorb onto particulate matter and accumulate in sediments, where they degrade at a rate which depends on environmental conditions and is difficult to assess. It should be emphasized that organotins deposited in sediments may undergo various processes such as resuspension, diffusion into the water column, or biotic and abiotic transformations. In all these conditions, they may pose a serious threat to aquatic organisms.

The problem of the presence of organotins in the marine environment has been highlighted in many international programs and conventions, e.g., concerning the Baltic Sea (Helsinki Commission (HELCOM)), the North-East Atlantic (OSPAR) or the Black Sea (BSC). Besides, tributyltin compounds are recognized as priority substances in the European Union (EU) directive on environmental quality standards in the area of the water framework policy (EU 2008). Finally, in 2008, the total ban on using harmful organotins in antifouling paints was introduced (EU 2003; IMO 2001). Nevertheless, the problem of OTs in the environment has not been totally solved. Since the total ban came into force, OTs contaminated sediments rather than vessels became a significant source of TBT and TPhT for aquatic life. According to the recommendations given by HELCOM, the available studies on TBT and TPhT in the Baltic Sea environment are insufficient, and more information about these harmful substances is needed. As far as the Southern Baltic Sea is concerned, published data are extremely scarce and relate mostly to ports and marinas (Falandysz et al. 2006; Filipkowska et al. 2011; Radke et al. 2008; Staniszewska et al. 2013).

The aim of this work was to assess the organotin contamination (tributyltin, triphenyltin, and their mono- and di-substituted degradation products) in sediments collected from three environmentally different basins of the Southern Baltic coastal zone: the Gulf of Gdańsk, and the Vistula (VL) and Szczecin Lagoons (SL). The samples were collected just after the implementation of the total ban on OTs in antifouling paints, to provide with the results of this work, a point of reference for assessing the effectiveness of the regulation in this region. We intended to go beyond the main ports of this region, where extremely high concentrations of organotins were recorded (Filipkowska et al. 2011), and study the essential factors affecting the distribution and fate of these compounds in an area covering a large range of environmental parameters (e.g., salinity, temperature, oxygen, depth).

## Material and methods

### Site description

Sediment samples were collected in three different basins located in the coastal zone of the Southern Baltic Sea: the Gulf of Gdańsk and the Vistula and Szczecin Lagoons.

The Gulf of Gdańsk (4,940 km^2^) is located in the south-eastern part of the Baltic Sea. The deepest part of this basin is the Gdańsk Deep (max. 118 m), whereas shallower waters are found in the Puck Bay (mean depth 3.1 m). The environment of the Gulf of Gdańsk is strongly determined by large inflows of fresh water from the Vistula River, the second largest river flowing into the Baltic Sea, and infrequent inflows of North Sea water through the Danish Straits. Both the Vistula River and the immediate proximity of the Tricity Agglomeration (Gdańsk, Sopot, and Gdynia) have an important influence on the environment of the Gulf of Gdańsk: input of nutrients and organic matter, two big international seaports (Gdańsk and Gdynia), marine traffic, and municipal pollution. This basin was proved to be a good area for studying the fate of pollutants in the marine environment (Lubecki and Kowalewska 2010).

The Vistula Lagoon (838 km^2^), separated from the Gulf of Gdańsk by the Vistula Spit, is shared between Poland and the Kaliningrad Region of Russia. It is a 91-km long basin with a maximum depth of 5.1 m (HELCOM 2003), where fresh water from numerous rivers and seawater from the Gulf of Gdańsk are continuously mixed (salinity 0.5–4.5). The Vistula Lagoon, which receives large inputs of nutrients from municipal and industrial wastewater and from agricultural run-off as well, has developed intense eutrophication symptoms (Glasby and Szefer 1998; HELCOM 2003). Moreover, oxygen deficiency may periodically appear in near-bottom waters, even though good oxygen conditions are normally observed in the water column (Szymczak-Żyła et al. 2011). There are many ports, fishing harbors, and marinas.

The Szczecin Lagoon (687 km^2^), shared by Poland and Germany, and located in the south-western part of the Baltic Sea, is the main part of the estuary of the Odra River, the second largest Polish river. It is a shallow (average depth 3.8 m) and low-salinity (from 0.5 to 2) basin (HELCOM 2003). The lagoon waters are exchanged with the Baltic Sea through three straits, but the inflow of water into the lagoon is dominated by the Odra River. This is a highly eutrophic basin, with high levels of nutrients and pollutants, enhanced levels of primary production, and oxygen depletion (Andrulewicz 1997; Szefer et al. 2009). The Szczecin Lagoon plays a significant role as a shipping route connecting the Port of Szczecin with the Port of Świnoujście. These ports form one of the biggest port groups in the Baltic Sea region.

### Sample collection

**Fig. 1:**
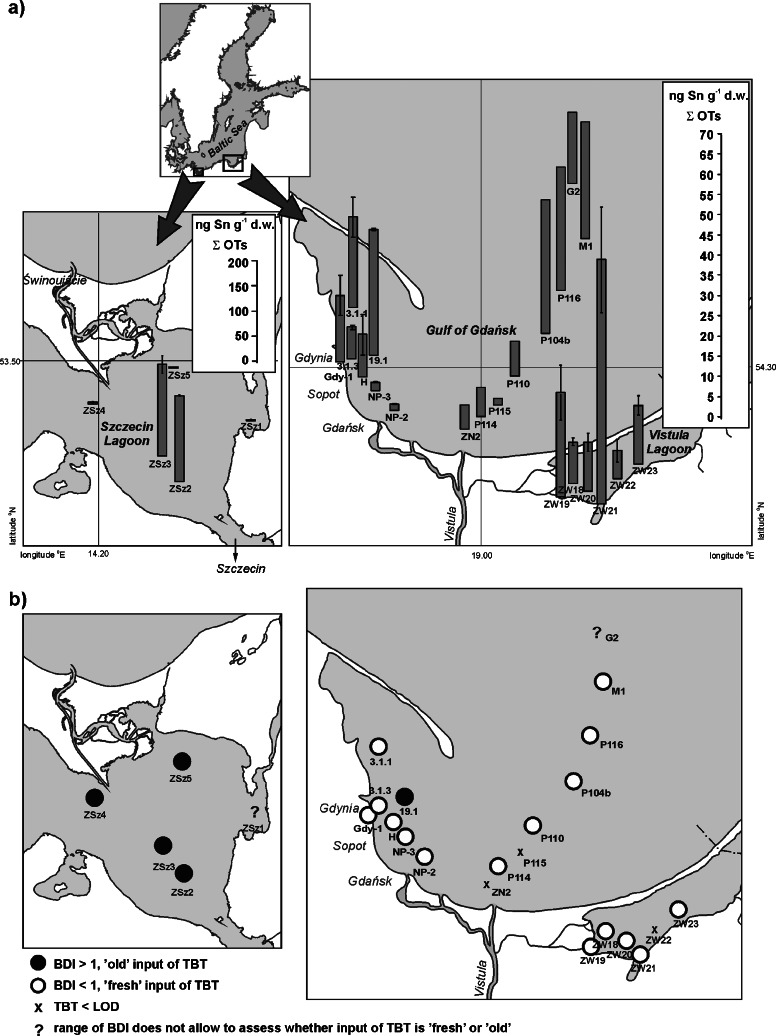
Concentrations of organotin compounds (**a**) and butyltin degradation indices (**b**) for sediments of the Gulf of Gdańsk, Vistula Lagoon and Szczecin Lagoon

Location of the sampling stations (Fig. 1a) was selected to represent both different levels of exposure to organotin pollution and varying environmental conditions.

The samples from the Gulf of Gdańsk were collected in 2008 along the coastline of the Tricity Agglomeration (3.1.1, 19.1, 3.1.3, Gdy-1, H, NP-3, NP-2) and also along the main spread of Vistula waters in this basin (ZN2-G2 profile: ZN2, P114, P115, P110, P104b, as far as the Gdańsk Deep: P116, M1, G2). The sediment samples were collected with a van Veen grab (0–5 cm), except for the stations of the ZN2-G2 profile, where a Niemistö core sampler (layers 0–1 and 1–5 cm) was used.

Samples from the Vistula Lagoon were collected in 2008, with a van Veen grab (0–5 cm). Six stations were selected on the Polish side of this basin (from ZW18 to ZW23). Three of them have distinctive features. Stations ZW19 and ZW21 are located in the river mouths, whereas station ZW23 represents a region near a fishing port.

The samples from the Szczecin Lagoon were collected in 2009, also with a van Veen grab collecting the very surface layer: 0–1 cm. Five stations were selected (from ZSz1 to ZSz5) and two of them (ZSz2 and ZSz3) are located close to the shipping route between two big Polish ports: Szczecin and Świnoujście.

Altogether, 26 sediment samples were collected and stored at −20 °C. The content of organotins, organic carbon, pigments, and also sediment grain size were determined in sub-samples.

### Organotin determinations

The following six OTs were analyzed in the sediment samples: TBT, dibutyltin (DBT), monobutyltin (MBT), TPhT, diphenyltin (DPhT), and monophenyltin (MPhT). OTs were quantified according to the procedure described by Bortoli et al. (2003), Pellizzato et al. (2004), and Filipkowska et al. (2011). Freeze-dried and homogenized sediment samples (0.6–2.1 g) were triply sonication-extracted with a methanol solution of tropolone with the addition of a small quantity of 37 % HCl and centrifuged. The combined extracts were then liquid/liquid partitioned in the system: extract/dichloromethane/NaCl solution (10 %), dewatered completely with activated Na_2_SO_4_, and then isooctane was added. Next step was a derivatization with a Grignard reagent (2.0-M pentylmagnesium chloride solution in tetrahydrofuran), followed by liquid/liquid extraction in the system: extract/n-hexane/H_2_SO_4_ solution (1 M), after which the internal standard (tripropylpentyltin) was added. To purify the extract, a column containing activated silica gel soaked with a mixture of n-hexane and toluene (1:1 (*v*/*v*)) was used, and organotin compounds were eluted with the same solution. The reduced extracts were injected into a gas chromatographic system (Varian 3900 GC, USA) coupled with a mass spectrometric detector (Saturn 2100T GC/MS, Varian). Determination of OT concentrations was based on the response factors derived from daily repeated injections of a standard mixture of derivatized compounds. The complete procedure is discussed in the cited papers.

The method was validated on the basis of an intercalibration between two laboratories (Table 1): Department of Environmental Sciences, University of Venice (DES, UNIVE), where the procedure was worked out and verified using the reference material CRM 477 (Bortoli et al. 2003; Pellizzato et al. 2004) and Marine Pollution Laboratory, Institute of Oceanology, Polish Academy of Sciences (MPL, IO PAS).

**Table 1:** Results of laboratory intercalibration [ng Sn g^−1^ d.w.] (Filipkowska et al. 2011)

Sediment sample	TBT	DBT	MBT	MPhT	DPhT	TPhT	∑ OTs
MPL, IO PAS *n* = 2	1,910 ± 70*	391 ± 13	165 ± 26	14 ± 2	<7	7 ± 1	2,490 ± 110
DES, UNIVE *n* = 2	1,570 ± 20	455 ± 4	207 ± 6	7 ± 3	<12	<3	2,240 ± 10

*Mean value ± *R*/2 (*R* =|*x*_1_–*x*_2_|)

### Additional analyses and measurements

#### Determination of organic carbon

Organic carbon concentration in sediments was determined using the wet chromic acid titration procedure according to the modified Walkley–Black method (Gaudette et al. 1974).

#### Grain-size characteristics

The grain-size characteristics of the sediments were determined according to the dry sieve method described by van Reeuwijk (2002) and supplemented with pipette analysis, as well as using the wet sieve analysis according to Folk and Ward (1957) or a laser size analyzer Mastersizer Micro Ver. 2.19.

#### Pigment analysis

Pigment determinations (chlorophyll-*a* and its derivatives) were carried out according to procedures described by Kowalewska (2005) and Szymczak-Żyła and Kowalewska (2007, 2011). Pigment analyses involved organic solvent extraction of the sediment followed by separation and identification using high performance liquid chromatography coupled to full spectrum diode array detectors.

#### Environmental parameters

During sediment sampling, the following parameters were measured: depth, temperature, salinity, and dissolved oxygen content in seawater. The measurements were carried out using a portable field meter (ProfiLine Multi 197i; WTW, Germany) and multiparametric sonde for water quality control (YSI 6000 UPG, USA; in the Szczecin Lagoon).

### Statistical analysis

The results were statistically processed using STATISTICA 6.0 software (StatSoft, Poland). The following methods were applied: Shapiro–Wilk normality test, the R-Spearman correlation analysis (as the data were not normally distributed in the vast majority of cases; *p* < 0.05 was regarded as significant) and principal component analysis (PCA), in order to evaluate the relationships between the organotin content in the sediment samples and other measured parameters.

## Results and discussion

### Concentrations of organotin compounds

**Fig. 2:**
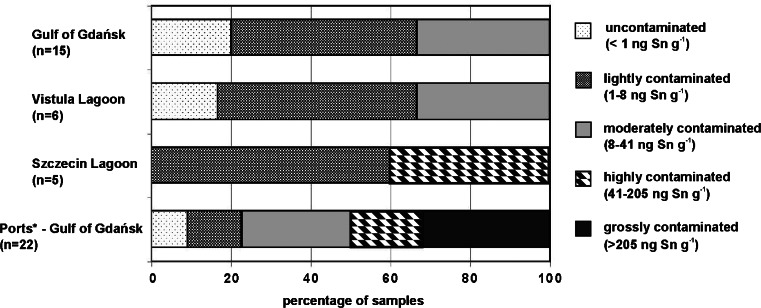
Classification of the sediment samples based on TBT concentration (according to Dowson et al. (1993); *Sediments from the ports were classified based on data given in Filipkowska et al. (2011))

Concentrations of organotins in the sediment samples from the Gulf of Gdańsk and the Vistula and Szczecin Lagoons ranged between 1 and 182 ng Sn g^−1^ d.w. (Fig. 1a). In these samples, only TBT and its degradation products (DBT and MBT) were found, whereas phenyltins were below the limit of detection (LOD) (Table 2). The highest levels were determined in the Szczecin Lagoon, at two stations located close to an important shipping route (Szczecin–Świnoujście): ZSz2, 171 ng Sn g^−1^ d.w., ZSz3, 182 ng Sn g^−1^ d.w. According to the classification suggested by Dowson et al. (1993), these sites were ranked as highly contaminated with TBT, whereas sediments from the Gulf of Gdańsk and Vistula Lagoon can be classified as moderately contaminated (Fig. 2). The highest butyltin contents in the samples from the Gulf of Gdańsk were about five times lower (stations: 19.1, P116, P104b—∑BTs ≈ 30 ng Sn g^−1^ d.w.) than in the Szczecin Lagoon, and two times lower than in the Vistula Lagoon (max. ∑BTs = 60 ng Sn g^−1^ d.w.—ZW21).

**Table 2:** Concentrations of TBT, DBT, and MBT (ng Sn g^−1^ d.w.), and butyltin degradation indices (BDI) in the sediment samples collected in the Gulf of Gdańsk, Vistula Lagoon, and Szczecin Lagoon

Station	TBT	DBT	MBT	BDI
Gulf of Gdańsk, along the coastline				
3.1.1	7.75 ± 1.52^a^	7.29 ± 1.43	7.26 ± 2.03	1.88
19.1	19.60 ± 0.46	6.65 ± 0.29	4.85 ± 0.12	0.59
3.1.3	3.12 ± 0.01	2.17 ± 0.20	2.44 ± 0.44	1.48
Gdy-1	6.82 ± 2.87	6.55 ± 1.73	3.06 ± 0.33	1.41
H	4.09 ± 2.67	3.38 ± 1.27	2.96 ± 1.03	1.55
NP-3	0.74 ± 0.15	0.80 ± 0.11	0.59 ± 0.11	1.88
NP-2	0.54 ± 0.08	0.51 ± 0.05	0.54 ± 0.01	1.94
Gulf of Gdańsk, ZN2-G2 profile^b^				
G2	9.6	5.4	2.5	0.81–1.14
M1	11.9	10.3	6.8	1.43
P116	13.7	8.2	8.6	1.22
P104b	10.9	11.1	10.9	2.03
P110	3.9	1.0	3.6	1.19–1.52
P115	1.5	<2.4^c^	<2.5^c^	ND
P114	3.6	<2.4^c^	3.6	1.01–1.68
ZN2	0.2	2.1	<3.2^c^	ND
Vistula Lagoon				
ZW18	2.02 ± 0.36	3.15 ± 0.33	5.10 ± 0.24	4.08
ZW19	10.82 ± 2.89	6.03 ± 2.22	9.15 ± 1.27	1.40
ZW20	4.30 ± 0.64	<4.8^c^	7.76 ± 2.83	1.80–2.92
ZW21	24.51 ± 4.67	11.83 ± 1.50	24.00 ± 6.92	1.46
ZW22	<5.7^c^	<5.5^c^	6.94 ± 2.86	ND
ZW23	5.33 ± 1.49	4.37 ± 0.51	4.79 ± 1.46	1.72
Szczecin Lagoon				
ZSz1	2.23 ± 0.18	<1.8^c^	<1.3^c^	<1.39
ZSz2	97.98 ± 6.08	38.66 ± 3.29	33.97 ± 1.50	0.74
ZSz3	115.2 ± 11.4	34.26 ± 4.51	32.28 ± 1.48	0.58
ZSz4	3.61 ± 1.00	<1.2^c^	<1.0^c^	<0.61
ZSz5	2.39 ± 0.24	<1.2^c^	<1.0^c^	<0.92

PhTs were not detected. LOD_TPhT_ = 2.2; LOD _DPhT_ = 7.3. LOD _MPhT_ = 2.5

*BDI* butyltin degradation index (MBT + DBT)/TBT

*ND* not calculated because values were below LOD

*TBT* tributyltin, *DBT* dibutyltin, *MBT* monobutyltin

^a^Mean value ± *R*/2 (*R* = |*x*_1_ – *x*_2_|);

^b^Weighted mean for 0–5-cm layer (obtained based on 0–1 and 1–5-cm layers)

^c^LOD—limit of detection (3× standard deviation of the blank)

Compared with the results obtained previously for two important ports of the Gulf of Gdańsk (Port of Gdańsk and Port of Gdynia) (Filipkowska et al. 2011), where many sites were ranked as severely contaminated with TBT (Fig. 2), with a peak organotin content of 19,180 ng Sn g^−1^ d.w., it seems that the maritime traffic on the studied basins had a definitely lower impact on sediment contamination than the activity of the ports. It confirms that since the IMO ban came into force, the main source of organotin compounds for aquatic life is the release from sediments settled in ports, harbors, and shipyards rather than from vessels. OTs in the most contaminated sediments will persist for years, and the risk of OT remobilization from sediments to the water phase is particularly high during dredging of shipping routes and port channels, and disposal of contaminated sediments at sea. That is why the results obtained for the Szczecin Lagoon give special cause for concern, as the main channel of this basin is artificially deepened. This activity worsens the risk for OT remobilization from sediments to the water phase and poses a threat to marine environment, especially to benthos and bottom fishes. In addition, even more contaminated sediments (deposited many years ago) can be uncovered during the dredging of the shipping route. In the case of sediments from the Vistula Lagoon where there is no channel for big ships, concentrations of OTs ranged between 7 and 60 ng Sn g^−1^ d.w.. This indicates that heavy traffic of small vessels and high amount of fishing nets, impregnated in the past with organotins and used for years in the study area, did not cause particularly high contamination of this basin.

As phenyltins were not detected in the sediments of the Gulf of Gdańsk and the Vistula and Szczecin Lagoons into which the two largest and many small Polish rivers flow in, it can be stated that phenyltin compounds were not significant ingredients of pesticides used in Poland. The fact that phenyltins were found only in the ports located on the Gulf of Gdańsk (Filipkowska et al. 2011; Radke et al. 2008) proves their origin from antifouling coating. In the case of butyltins, no other significant sources of DBT and MBT, except for TBT, were found in the study area. Predominance of either DBT or MBT, combined with high concentrations of these compounds, was not observed. Moreover, highly positive correlation coefficients between contents of TBT and DBT (0.78–0.99, *p* < 0.05), and also TBT and MBT (0.78–0.98, *p* < 0.05) recorded in all studied basins, can be explained as the effect of TBT degradation processes.

Comparison of butyltin levels in the sediments of this study with the results obtained by researchers in different parts of the world (see Supplementary Table—Online Resource) shows a relatively low butyltin contamination of the Southern Baltic coastal zone. However, it should be emphasized that published data on the OTs in sediments concern mostly ports, harbors, shipyards, or marinas. This work includes also samples in areas much less affected by this kind of anthropogenic stress.

### Degradation index of butyltin compounds

Degradation of organotins is caused by various processes. However, in the marine environment, in particular on the sea bed, biological cleavage is the most important one. There is evidence that some microorganisms, like bacteria (e.g., Pseudomonads, *Alcaligenes faecalis*, *Shewanella putrefaciens*) and phytoplankton (e.g., *Skeletonema costatum*, *Chlorella vulgaris*, *Scenedesmus dimorphus*), have the ability to degrade organotin compounds (Hoch 2001; Lee et al. 2012; Sampath et al. 2012; Tam et al. 2002). As a consequence, the environmental conditions determining the growth of these microorganisms, such as pH, temperature, oxygen, turbidity, and light, are also factors recognized as responsible for the degradation of these contaminants. The TBT half-life in the marine environment is highly variable. In seawater, it is estimated to range between a few days and a few weeks (Stewart and de Mora 1990), whereas in sediments, between a few months and several dozen years (Dowson et al., 1996; Takeuchi et al., 2004; Watanabe et al., 1995). Many studies have shown that aerobic biodegradation is faster than the anaerobic one (Antizar-Ladislao 2008; Blunden and Evans 1990; Gadd 2000). The rate of OT degradation depends not only on the sediment type and chemical species (e.g., chlorides, oxides, hydroxides), but also on the OTs concentration itself, as decomposition processes are inhibited if high concentrations of TBT accumulated in sediments (Dowson et al. 1996; Hoch 2001; Stewart and de Mora 1990). Moreover, the persistence of TBT increases when it is associated with paint particles (Page et al. 1996; Thomas et al. 2000). For all these reasons, it is not easy to determine how recent the input of OTs into sediments is. However, based on the butyltin degradation index (BDI), estimation can be attempted. BDI is the most commonly used degradation index, defined as the ratio between the sum of concentrations of the two main degradation products (MBT and DBT) and that of the parent compound (TBT) (Díez et al. 2002). The values of BDI for the samples are shown in Table 2. As BDI values are higher than 1 for the samples collected from the Gulf of Gdańsk (1.22–2.03) and Vistula Lagoon (1.40–4.08), it can be stated that TBT input into sediments of these basins is “old.” There is only one exception, namely station 19.1, where the BDI value is 0.59 indicating “fresh” input of TBT. It is worth noting that station 19.1 is located between the dumping site and anchorage belonging to the Port of Gdynia, where also a previous study provided evidence of a “fresh” input of TBT (Filipkowska et al. 2011). Moreover, the sediment sample from this station was the most contaminated among those collected along the coastline of the Tricity. All these remarks show that the sediment disposal sites of the Gulf of Gdańsk are still an important source of OTs for marine environment: dredged material from the ports is routinely discharged into these sites without any monitoring of sediments for organotin compounds. A relatively recent input of TBT recognized in the Szczecin Lagoon also gives special cause for concern. A sorting of the sediment samples showing “fresh” and “old” inputs of the parent compound is presented in Fig. 1b.

### The role of the environmental conditions

**Fig. 3:**
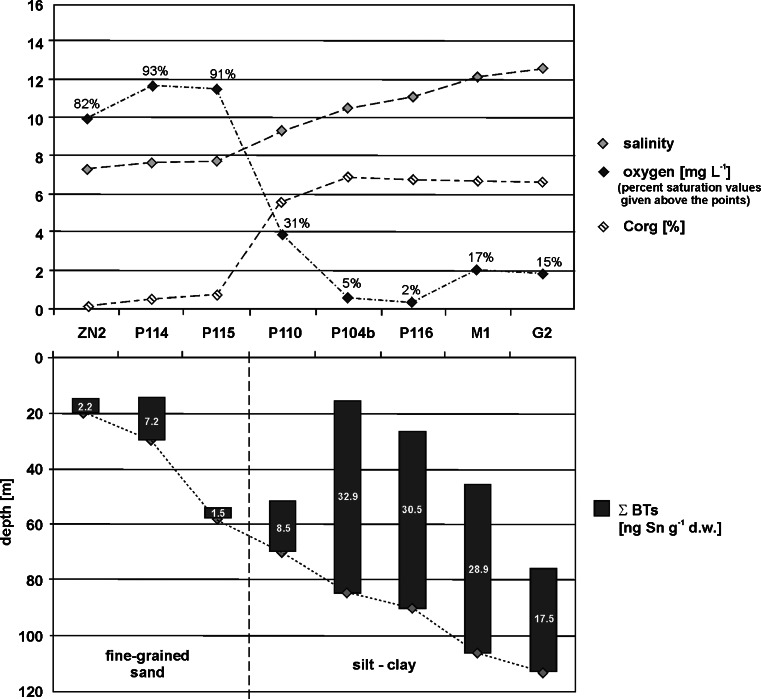
Concentrations of BTs in sediments of the Gulf of Gdańsk against the background of environmental parameters of the near-bottom seawater (salinity, dissolved oxygen content) and sediments (type, organic carbon content), along the profile ZN2-G2

Degradation indices provide valuable information, but it is essential to consider also the prevailing environmental conditions in the study sites to avoid misinterpreting the BDI values. Similar values of BDI obtained for (1) a sandy sediment sample taken from a site with good oxygen conditions (e.g., station P114) and (2) a clayey sediment taken from a site with oxygen depletion (e.g., station M1), do not prove that the period of OT accumulation in study sediments was long the same. Apart from environmental conditions, it is also worth considering the relative abundance of individual TBT breakdown products. Then, it can be seen, for example, that the longer the distance from the Vistula outlet, the lower the percentage of MBT in the sum of butyltins (BTs). The high negative correlation coefficients between percentage of MBT in sediments and seawater salinity (−0.74, *p* < 0.05) or water depth (−0.74, *p* < 0.05) also demonstrate this relationship and indicate that environmental conditions along the profile ZN2-G2 are more and more unfavorable to degradation of butyltins. Figure 3 shows how much the environmental conditions change in the study area. Station P110 seems to be a turning point in the ZN2-G2 profile: seawater salinity near the bottom increase, oxygen deficiency appears (<4 mg O_2_ L^−1^) followed by severe oxygen deficiency (<2 mg O_2_ L^−1^) at the stations from P104b to G2, sediment type changes and the content of organic carbon increases. It seems that dissolved oxygen depletion in deep bottom waters is the key factor enhancing the persistence of butyltins in sediments of the Gdańsk Deep, as it was proved by highly negative correlation coefficients between butyltins and dissolved oxygen (from −0.81 to −0.88, *p* < 0.05) in the profile ZN2-G2. For the reasons of oxygen depletion and increase in the water column depth, the growth of benthic species is limited and the number of aerobic bacteria capable of OT degradation decreases. All these factors extend the time of BT accumulation in the sediments of Gdansk Deep, and highly positive correlation coefficients between concentrations of organic carbon and butyltins (0.93, *p* < 0.05) further support the thesis that organotins are adsorbed onto particulate organic matter (Berg et al. 2001; Hoch and Schwesig 2004). Due to the fact that sunlight does not penetrate into the Gdańsk Deep, the organic matter deposited there with sorbed BTs is protected from photodegradation; this is confirmed by the high concentrations of chlorophyll-*a* (42.3–80.6 nmol g^−1^), which is a highly unstable compound (Szymczak-Żyła and Kowalewska 2007). It should be emphasized that despite unfavorable conditions for degradation of TBT, which prevail in the Gdańsk Deep, the average percentage of breakdown products of TBT is as much as 56 %, which may be caused by a very long time of residence of butyltins in the sediments.

Environmental conditions quite different from those in the Gulf of Gdańsk are characteristic of the sampling sites located in the VL and SL: shallow waters (VL 2–3 m; SL 2–5 m), low salinity (VL 0.3–2.3; SL 1.3–1.7), good oxygen conditions (VL 7.0–11.8 mg O_2_ L^−1^, i.e., 75–135 % O_2_ saturation (WIOŚ, 2010); SL 7.7–10.8 mg O_2_ L^−1^, i.e., 77–110 % O_2_ saturation). Although the lagoons are environmentally similar, the greatest differences in OT degradation rate were observed between these two basins. In the Vistula Lagoon, degradation products of TBT dominated (mean 29 % TBT in the sum of BTs), whereas in the Szczecin Lagoon, considerable predominance of TBT was recorded (mean 84 % TBT in the sum of BTs). Human activities in both the lagoons are very different, and both percentages of individual butyltins and their concentrations were observed to be different as well. In these cases, differences in the rate of OT degradation are not a result of more or less favorable environmental conditions: this was confirmed by the fact that statistically significant correlations between percentage of individual butyltins and parameters of the near-bottom water were not found. For the Vistula and Szczecin Lagoons, the most significant differentiating factor is the elapsed time of BT input into sediments, and BDI seems to be a highly appropriate tool for comparing BT degradation rates. In both lagoons, as well as in the Gulf of Gdańsk, highly positive correlation coefficients were observed between concentrations of BTs and organic carbon (0.82–0.99, *p* < 0.05), chloropigments-*a* (0.86–0.87, *p* < 0.05), and percentage of fine sediment particles (*Ø* <0.063 mm) (0.77–0.93, *p* < 0.05). This indicates that phytoplankton plays an important role in the transport of butyltin compounds from the water column to the bottom sediments. An exception was the lack of correlations between butyltins and chloropigments-*a* in the samples collected along the coastline of the Tricity Agglomeration. This area is quite special because of the short distance from two big international ports, including anchorages and dumping sites, and also the relatively favorable environmental conditions for degradation of organotin compounds (8–17-m depth; 8.7–9.1 mg O_2_ L^−1^, i.e., 70–86 % O_2_ saturation; 0.06–2.09 % Corg). In this case, the differences in concentration/percentage of TBT and its derivatives may arise from the short distance from potential sources of butyltins.

**Fig. 4:**
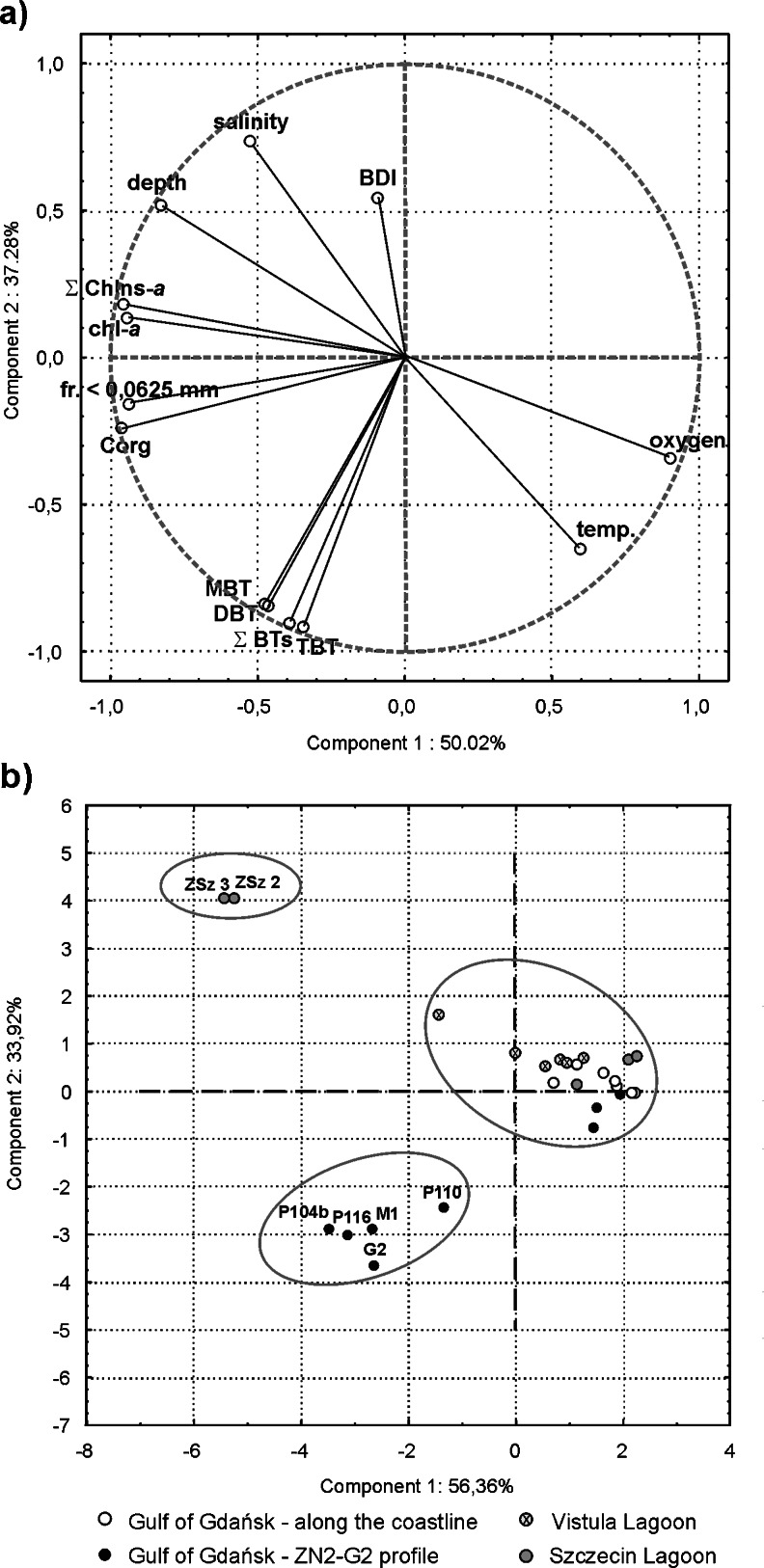
Scatter plot of **a** principal component loadings by individual variables and **b** principal component object scores by sampling sites, based on BT concentrations, organic carbon, and pigment contents, parameters of the near-bottom seawater, and percentage of the fine sediment particles

The results of the correlation analysis between BTs, environmental parameters, and pigments were confirmed by a principal component analysis (Fig. 4a). This statistical method was applied to verify the obtained results and find the most significant factors affecting the fate of organotins in the study area. The PCA data matrix model explains 87 % of the total variance with the first two principal components, and represents well almost all the variables. The first principal component (50 % of the total variance) distinguishes a large group of variables containing all BTs, organic carbon, pigments, the <0.063-mm grain-size fraction, together with parameters like water depth and salinity of the near-bottom layer, thus confirming a high, positive correlation between these variables. The negative, quite high loadings of oxygen content and temperature indicate that as the values of these parameters increase, the butyltin content and organic matter decreases. It should be emphasized that some of these relationships may be a secondary effect, as some parameters like depth, temperature, dissolved oxygen content, and salinity of the bottom water are strongly related in the Gulf of Gdańsk. Basing on the second principal component (37 % of the total variance) one may expect that with increasing BT concentrations, the value of butyltin degradation index decreases. Both principal components pointed out a compact group composed of individual BTs and their sum, thus confirming a high, positive correlation between these compounds. This can be explained as the effect of similar sources of organotins and similar accumulation and degradation processes in the study environment. The results of principal component analysis are in agreement with the existing knowledge of the impact of environmental conditions on the fate of butyltins in the environment (Berg et al. 2001; Hoch 2001; Hoch and Schwesig 2004; Stewart and de Mora 1990). This analysis showed that water temperature may also have an impact on degradation processes of butyltins in sediments. This corresponds also to the conclusions presented by Negri and Marshall (2009), who compared organotin pollution in the Great Barrier Reef and Antarctica; the authors pointed out that the degradation of organotins is much slower in a colder environment.

Additionally, the principal component analysis was applied to show the diversity of sampling stations (Fig. 4b). On the score plot defined by the two principal components (90 % of the total variance explained), three groups of stations are separated: the first one contains stations located along the shipping route in the Szczecin Lagoon, the second groups together deep stations from the profile ZN2-G2 of the Gulf of Gdańsk (from P110 to G2), and the last gathers the rest of the sampling stations. When considering components separately, Component 1 seems to be largely related to the butyltin content in sediments, whereas Component 2 may be associated with salinity of the near-bottom water.

## Conclusions

This study assesses the organotin contamination of the sediments in three different basins of the Southern Baltic coastal zone: the Gulf of Gdańsk and the Vistula and Szczecin Lagoons. Butyltins were found in all the sediment samples and their concentrations were different depending on the sampling location: the highest were in the Szczecin Lagoon, along the shipping route Szczecin–Świnoujście, where recent tributyltin input was observed. Sediments from the Gulf of Gdańsk and Vistula Lagoon were less contaminated with butyltin compounds and rich in degradation products of tributyltin, except for sediment collected close to the Gdynia anchorage and dumping site. Phenyltins were not detected in any of the sediment examined.

Correlations between organotin concentrations in sediments and environmental parameters were high and significant. Oxygen deficiency is a main factor that extends the residence time of OTs in sediments. Moreover, the persistence of butyltin compounds in sediments of the Southern Baltic Sea can be enhanced by the following factors: high salinity, low temperature, high water column depth, high organic matter content, and high percentage of fine grain-size fraction. Favorable conditions for OT degradation were observed near the coastline of the Gulf of Gdańsk, whereas in the Deep of Gdańsk, the environmental conditions favor a longer persistence of these compounds.

Despite the total ban on using harmful organotins in antifouling paints on ships, butyltin compounds are still present in the sediments of the Southern Baltic coastal zone. High concentrations of butyltins in sediments adjacent to continuously dredged shipping routes and dumping sites, pose a significant threat to marine life, especially if we consider that in Polish regulations there are neither concentration limits nor monitoring obligation for organotins in dredged materials.

## Electronic supplementary material

Below is the link to the electronic supplementary material.

ESM 1 (PDF 61 kb)
