# Supplementary material for: Organotin compounds in surface sediments of the Southern Baltic coastal zone: a study on the main factors for their accumulation and degradation
Source: Environ Sci Pollut Res Int. 2013 Sep 12;21(3):2077–87. doi: 10.1007/s11356-013-2115-x (PMC3906560; doi:10.1007/s11356-013-2115-x)
Supplement: Supplementary file 1 — (PDF 61 kb) [file 11356_2013_2115_MOESM1_ESM.pdf]

Anna Filipkowska, Grażyna Kowalewska, Bruno Pavoni

ORGANOTIN COMPOUNDS IN SURFACE SEDIMENTS OF THE SOUTHERN BALTIC COASTAL ZONE: A STUDY ON THE MAIN FACTORS FOR THEIR ACCUMULATION AND DEGRADATION

Environmental Science and Pollution Research

Anna Filipkowska, Grażyna Kowalewska
Marine Pollution Laboratory, Institute of Oceanology, Polish Academy of Sciences, ul. Powstańców Warszawy 55, 81-712 Sopot, Poland

Bruno Pavoni
Department of Environmental Sciences, Informatics and Statistics, University of Venice, Calle Larga S. Marta 2137, 30123 Venice, Italy

Corresponding author: Anna Filipkowska, e-mail: afilipkowska@iopan.gda.pl

Supplementary Table. Concentration of organotin compounds in sediments from different areas around the world

Location	Date of sampling	Sediment layer [cm]	Concentration range [ng Sn g^{-1} d.w.]				References
			TBT	DBT	MBT	PhTs	
Europe							
Baltic Sea, Poland:<br>Port of Gdańsk	1998	0-20	1 066 – 16 400*	1 020 – 21 420*	816 – 31 280*	-	Senthilkumar et al. 1999
	2003, 2005	0-25	86 – 7 635	49 – 1 293	22 – 411	TPhT: <4 – 15<br>DPhT: <6 – 22<br>MPhT: <7– 46	Radke et al. 2008
	2008	0-5	13 – 15 775	9 – 2 060	7 – 684	TPhT: max. 339<br>MPhT: max. 320	Filipkowska et al. 2011

Port of Gdynia	2008	0-5	8 – 1 914	5 – 391	3 – 165	TPhT: max. 14<br>MPhT: max. 18	Filipkowska et al. 2011
	2009	0-25	1 143 – 6 408	250 – 2 716	134 – 968	-	Radke et al. 2012
marinas of Gdańsk<br>marinas of Gdynia	1998	-	20 – 5 800<br>10 – 9 600	20 – 2 700<br>10 – 5 000	100 – 6 600<br>110 – 5 600	-<br>-	Falandysz et al. 2002
Gulf of Gdańsk, coast, ports	2002-2003	0-10	ΣBTs: n.d. – 30 000			-	Falandysz et al. 2006
Baltic Sea, Germany:<br>marinas/harbours	1997-1998	-	234 – 6 970*	77 – 7 140*	7 – 884*	TPhT: <6 – 1 292*<br>DPhT: n.d. – 148*<br>MPhT: n.d. – 37*	Biselli et al. 2000
Baltic Sea, Sweden:<br>ports (e.g. Stockholm)	2006-2007	0-2	27 – 533*	19 – 714*	24 – 673*	-	Eklund et al. 2010
Baltic Sea, Kattegat Strait:<br>Port of Göteborg	2000	0-2	18 – 118*	11 – 46*	4 – 30*	TPhT: <0.5 – 24*<br>DPhT: <0.5 – 3*<br>MPhT: <0.5 – 3*	Brack 2002
North Sea, Norway:<br>Port of Oslo<br>Port of Drammen	2005	0-5	172 – 779*<br>123 – 1 107*	61 – 168*<br>31 – 153*	42 – 54*<br>10 – 29*	-<br>-	Cornelissen et al. 2008
North Sea, Germany:<br>marinas/harbours	1997-1998	-	33 – 295*	15 – 143*	7 – 41*	TPhT: <6 – 31*<br>DPhT: n.d. – 17*<br>MPhT: <6*	Biselli et al. 2000
North Sea, Netherlands:<br>Wadden Sea coast	1998	-	max. 252*	max. 48*	max. 12*	TPhT: max. 13*<br>DPhT: max 7*<br>MPhT: max. 4*	Van den Brink<br>and Kater 2006
Atlantic Ocean, Portugal:<br>coast	1999-2000	0-2	2 – 5*	<3 – 33*	<3 – 53*	-	Díez and Bayona 2009

Nazaré canyon	2005-2006	0-6	<0.1 – 470	0.5 – 340	<5 – 370	TPhT: <0.1 – 40<br>DPhT: <0.1 – 3.3	Sousa et al. 2012
Mediterranean Sea, Spain:<br>ports, north-eastern coast	1995	0-2	max. 7 676*	max. 3 499*	max. 1 138*	TPhT: max 80*<br>DPhT: max 182*<br>MPhT: max 3 148*	Díez et al. 2002
ports, south-eastern coast	1999-2000	0-2	max. 1 586*	max. 415*	max. 534*	TPhT: max 92*<br>DPhT: max 59*<br>MPhT: max 85*	
Barcelona harbour	2002	0-5	98 – 4 702	67 – 2 607	35 – 440	-	Díez et al. 2006
Mediterranean Sea, France:<br>marinas/ports	2004	0-10	37 – 4 402	34 – 3 025	65 – 3 682	TPhT: 3 – 358<br>DPhT: <2 – 124	Cassi et al. 2008
Adriatic Sea, Italy:<br>Venice Lagoon	2003	0-2	21 – 39 300	2 – 12 623	-	-	Berto et al. 2007
Asia							
Indian Ocean:<br>Persian Gulf, Gulf of Oman	2000-2001	0-2	<0.1 – 60	<0.1 – 30	<0.1 – 10	-	de Mora et al. 2003
Indian Ocean, Arabia Sea, India:<br>Kochi harbour<br>Mumbai harbour	2000-2002<br>2000-2001	-<br>-	7 – 6 895*<br>2 – 489*	n.d. – 239*<br>n.d. – 67*	-<br>-	-<br>-	Bhosle et al. 2006
Pacific Ocean, China:<br>Port of Xiamen	2006	-	max. 26	max. 3,4	max. 115	TPhT: max. 1<br>DPhT: max. 16<br>MPhT: max. 1,1	Wang et al. 2008
Pacific Ocean, Taiwan:<br>international ports<br>fishing ports	2001<br>2001-2004	-	35 – 555*<br>1 – 3 505*	-<br>-	-<br>-	-<br>-	Lee et al. 2006

Pacific Ocean, Japan: Otsuchi Bay, ports	2005	-	<0.4 – 5 740*	1 – 1 734*	<0.7 – 2 244*	TPhT: <0.3 – 1 190*<br>DPhT: <0.4– 300*<br>MPhT: <0.6 – 3 172*	Harino et al. 2007
Pacific Ocean, Philippines: Manila Bay	2005	-	0.5 – 9	0.7 – 17	3.1 – 49	TPhT: < 0.5<br>DPhT: < 0.8	Olivares et al. 2013
Africa							
Mediterranean Sea, Tunisia: Bizerte Lagoon	1999-2000	0-10 (20)	ΣBTs: max. 170			max. 55	Mzoughi et al. 2005
Indian Ocean, Tanzania:<br>Port of Zanzibar<br>Port of Dar es Salaam	2004	-	max. 3 670<br>max. 16 700	20 – 63 600<br>1 – 79 300	1 – 976<br>max. 5900	-<br>-	Sheikh et al. 2007
North America							
Atlantic Ocean, Canada, Gulf of Saint Lawrence: Saguenay Fjord	2001-2002	-	ΣBTs: 6 – 288			-	Viglino et al. 2004
Pacific Ocean, Mexico: Port of Ensenada	1995	-	ΣBTs: 33 – 1 021			-	Macias-Carranza et al. 1997
South America							
Atlantic Ocean, Brazil: Guanabara Bay, ports	-	0-5	10 – 521	<4.5 – 395	<4.7	TPhT: <3.9 – 39<br>DPhT: <4.9<br>MPhT: <4.5 – 19	Fernandez et al. 2005
Atlantic Ocean, Argentina: Port of Belgrano	2004-2006	0-10	max. 3 288	max. 1 645	-	-	Delucchi et al. 2007

Pacific Ocean, Chile: San Vincente Bay	2006	0-10	14 – 1 560	<20 – 1 170	<27 – 470	-	Pinochet et al. 2009
Australia							
Pacific Ocean, Australia, Great Barrier Reef:	1999	0-10					Haynes and Loong 2002
marinas			<1 – 5.5	-	-	-	
ports			<1 – 1 275	-	-	-	
shipgrouding site			7 500 – 340 000	660 – 32 000	660 – 61 000	-	
Pacific Ocean, Australia: south-east Queensland, marina	-	0-20	90 – 3 587*	76 – 2 779*	88 – 2 890*	-	Burton et al. 2005
Antarctica							
Southern Ocean, Ross Sea	-	0-1	<20 – 2 110	<20 – 180	<20 – 28	-	Negri et al. 2004

* – values converted into ng Sn g^{-1}, n.d. – not detected, TBT – tributyltin, DBT – dibutyltin, MBT – monobutyltin, BTs – butyltins, TPhT- triphenyltin, DPhT – diphenyltin, MPhT – monophenyltin, PhTs – phenyltins,

References

Berto D, Giani M, Boscolo R, Covelli S, Giovanardi O, Massironi M, Grassia L (2007) Organotins (TBT and DBT) in water, sediments, and gastropods of the southern Venice lagoon (Italy). Mar Pollut Bull 55:425–435

Bhosle NB, Garg A, Harji R, Jadhav S, Sawant SS, Krishnamurthy V, Anil C (2006) Butyltins in the sediments of Kochi and Mumbai harbours west coast of India. Environ Int 32:252–258

Biselli S, Bester K, Hühnerfuss H, Fent K (2000) Concentrations of the antifouling compound Irgarol 1051 and of organotins in water and sediments of German North and Baltic Sea marinas. Mar Pollut Bull 40:233–243

Brack K (2002) Organotin compounds in sediments from the Göta Älv estuary. Water Air Soil Pollut 135:131–140

Burton ED, Phillips IR, Hawker DW (2005) In-situ partitioning of butyltin compounds in estuarine sediments. Chemosphere 59:585–592

Cassi R, Tolosa I, de Mora S (2008) A survey of antifoulants in sediments from Ports and Marinas along the French Mediterranean coast. Mar Pollut Bull 56:1943–1948

Cornelissen G, Pettersen A, Nesse E, Eek E, Helland A, Breedveld GD (2008) The contribution of urban runoff to organic contaminant levels in harbour sediments near two Norwegian cities. Mar Pollut Bull 56:565–573

Delucchi F, Tombesi NB, Freije RH, Marcovecchio JE (2007) Butyltin compounds in sediments of the Bahía Blanca Estuary, Argentina. Environ Monit Assess 132:445–451

de Mora SJ, Fowler SW, Cassi R, Tolosa I (2003) Assessment of organotin contamination in marine sediments and biota from the Gulf and adjacent region. Mar Pollut Bull 46:401–409

Díez S, Ábalos M, Bayona JM (2002) Organotin contamination in sediments from the Western Mediterranean enclosures following 10 years of TBT regulation. Water Res 36:905–918

Díez S, Jover E, Albaigés J, Bayona JM (2006) Occurrence and degradation of butyltins and wastewater marker compounds in sediments from Barcelona harbor, Spain. Environ Int 32:858–865

Díez S, Bayona JM (2009) Butyltin occurrence and risk assessment in the sediments of the Iberian Peninsula. J Environ Manage 90:S25–S30

Eklund B, Elfström M, Gallego I, Bengtsson B-E, Breitholtz M (2010) Biological and chemical characterization of harbour sediments from the Stockholm area. J Soils Sediments 10:127–141

Falandysz J, Brzostowski A, Szpunar J, Rodriguez-Pereiro I (2002) Butyltins in sediments and three-spined stickleback (*Gasterosteus aculleatus*) from the marinas of the Gulf of Gdańsk, Baltic Sea. J Environ Sci Heal A 37:353–363.

Falandysz J, Albanis T, Bachmann J, Bettinetti R, Bochentin I, Boti V, Bristeau S, Daehne B, Dagnac T, Galassi S, Jeannot R, Oehlmann J, Orlikowska A, Sakkas V, Szczerski R, Valsamaki V, Schulze-Oehlmann U (2006) Some chemical contaminant of surface sediments at the Baltic Sea coastal region with special emphasis on androgenic and anti-androgenic compounds. J Environ Sci Heal A 41:2127–2162

Fernandez MA, de Luca Rebello Wagener A, Limaverde AM, Scofield AL, Pinheiro FM, Rodrigues E (2005) Imposex and surface sediment speciation: A combined approach to evaluate organotin contamination in Guanabara Bay,Rio de Janeiro, Brazil. Mar Environ Res 59:435–452

Filipkowska A, Kowalewska G, Pavoni L, Łęczyński L (2011) Organotin compounds in surface sediments from seaports on the Gulf of Gdańsk (southern Baltic coast). Environ Monit Assess 182:455–466

Harino H, Yamamoto Y, Eguchi S, Kawai S, Kurokawa Y, Arai T, Ohji M, Okamura H, Miyazaki N (2007) Concentrations of antifouling biocides in sediment and mussel samples collected from Otsuchi Bay, Japan. Arch Environ Contam Toxicol 52:179–188

Haynes D, Loong D (2002) Antifoulant (butyltin and copper) concentrations in sediments from the Great Barrier Reef World Heritage Area, Australia. Environ Pollut 120:391–396

Lee C-C, Hsieh C-Y, Tien C-J (2006) Factors influencing organotin distribution in different marine environmental compartments, and their potential health risk. Chemosphere 65:547–559

Macias-Carranza VA, Macias-Zamora JV, Villaescusa-Celaya JA (1997) Organotin compound in marine water and sediments from the port of Ensenada, Baja California, Mexico. Ciencias Marinas 23:377–394

Mzoughi N, Lespes G, Bravo M, Dachraoui M, Potin-Gautier M (2005) Organotin speciation in Bizerte lagoon (Tunisia). Sci Tot Environ 349:211–222

Negri AP, Hales LT, Battershill C, Wolff C, Webster NS (2004) TBT contamination identified in Antarctic marine sediments. Mar Pollut Bull 48:1142–1144

Olivares RU, Tabeta S, Sombrito EZ (2013) Tributyltin in marine sediments and Philippine green mussels (Perna viridis) in Manila Bay. J Mar Sci Technol 18:213–219

Pinochet H, Tessini C, Bravo M, Quiroz W, de Gregori I (2009) Butyltin compounds and their relation with organic matter in marine sediments from San Vicente Bay - Chile. Environ Monit Assess 155:341–353

Radke B, Łęczyński L, Wasik A, Namieśnik J, Bolałek J (2008) The content of butyl- and phenyltin derivatives in the sediment from the Port of Gdańsk. Chemosphere 73:407–414

Radke B, Wasik A, Jewell LL, Piketh S, Pączek U, Gałuszka A, Namieśnik J (2012) Seasonal changes in organotin compounds in water and sediment samples from the semi-closed Port of Gdynia. Sci Tot Environ 441: 57–66

Senthilkumar K, Duda CA, Villeneuve DL, Kannan K, Falandysz J, Giesy JP (1999) Butyltin compounds in sediment and fish from the Polish coast of the Baltic Sea. Environ Sci Pollut Res 6:200–206

Sheikh MA, Noah NM, Tsuha K, Oomori T (2007) Occurrence of tributyltin compounds and characteristics of heavy metals. Int J Environ Sci Technol 4:49–59

Sousa ACA, Oliveira IB, Laranjeiro F, Takahashi S, Tanabe S, Cunha MR, Barroso CM (2012) Organotin levels in Nazaré canyon (west Iberian Margin, NE Atlantic) and adjacent coastal area. Mar Pollut Bull 64:422–464

Van den Brink PJ, Kater BJ (2006) Chemical and biological evaluation of sediments from the Wadden Sea, the Netherlands. Ecotoxicology 15:451–460

Viglino L, Pelletier É, St-Louis R (2004) Highly persistent butyltins in northern marine sediments: A longterm threat for the Saguenay Fjord (Canada). Environ Toxicol Chem 23:2673–2681

Wang X, Hong H, Zhao D, Hong L (2008) Environmental behavior of organotin compounds in the coastal environment of Xiamen, China. Mar Pollut Bull 57:419–424
